# Multivalent glycosystems for nanoscience

**DOI:** 10.3762/bjoc.10.244

**Published:** 2014-10-08

**Authors:** Thisbe K Lindhorst

**Affiliations:** 1 Otto Diels Institute of Organic Chemistry, Christiana Albertina University of Kiel, Otto-Hahn-Platz 3/4, 24098 Kiel, Germany

**Keywords:** multivalent glycosystems

Carbohydrates constitute the most abundant class of biomolecules on Earth. They are “global players” and indeed play many roles. They store energy and water. They serve as metabolic intermediates. They function as stable, raw materials or as intelligent, bioengineered products. They are even active in “social media”, that is, they mediate communication between cells and they store information by molecular shaping. They adopt more forms and shapes than one can conceive in 24 hours. They exist as small mono- or oligo-saccharides, as large and highly complex polysaccharides, and in conjugation with, for example, proteins and lipids, forming a kingdom of glycoconjugates that are found on every cell surface.

Hence, carbohydrates are like Alice’s Wonderland—they can assume any role. They can be big or small, sweet or sour, silent or explicit, crucial or, less often, irrelevant. What we do understand, however, is that carbohydrates are essential to life, both in a state of health and in the case of disease. Consequently, the glycosciences have gained great respect in chemistry and biology. It is now generally considered important to be able to synthesize carbohydrates and glycoconjugates, to purify and analyze their structures, and to advance our understanding of the biology of carbohydrates in living systems. The two Thematic Series “Synthesis in the glycosciences” I [1] and II [2–35] that precede this Thematic Series have impressively documented the state of the art in this field of research.

Now, a third “sweet” Thematic Series is presented in which the borderline between Alice’s Wonderland and real-life applications of carbohydrates has been deliberately crossed. The structural world of natural sugars has been extended towards artificial carbohydrate architectures to achieve potential innovations offered by glycoconjugates. Since 2011, approximately 70 group leaders from 21 countries have gathered in an international COST (European Cooperation in Science and Technology) Action, funded by the European Union (EU) to address the challenges and opportunities in the field of glycosciences. This enterprise has focused its research on the potential applications and social benefits that lie in the field. For instance, multivalent glycoconjugates can be used as anti-adhesive drugs against microbial infections. They could be developed into bioimaging agents that can target specific tissues. Indeed, they will certainly find applications in materials science and in medicine, for example, in drug delivery or diagnostics [36]. Consequently, this COST Action (CM1102) [37] was called “MultiGlycoNano” to illustrate that multivalent glycostructures now enter the era of nanoscience. Some of the achievements of this team of researchers are collected within the presented volume.

I am very grateful to the five colleagues of our international network who have dealt with the manuscripts submitted for this Thematic Series entitled, “Multivalent glycosystems for nanoscience”. Bruce Turnbull (United Kingdom) has amassed contributions dedicated to “Synthetic methods to make multivalent glycosystems”, Alessandro Casnati (Italy) has delivered manuscripts regarding “Glycoconjugates for the delivery of drugs and diagnostic probes” for review, Roland Pieters (The Netherlands) has taken care of manuscripts related to “Glycoconjugates as anti-pathogenic agents”, Vladimir Křen (Czech Republic) has supervised the area of “Glycoconjugates as modulators of the immune system”, and finally, Jean-Louis Reymond (Switzerland) has received contributions regarding “Glycosystems in nanotechnology”. As a result, 27 excellent publications are presented in this fine collection. I would like to sincerely congratulate all contributors for their research and results. We can be proud of what has been achieved. We should be inspired by what has been suggested. And we have definitely been encouraged by the delightful experiences we shared in an international network of colleagues and friends. In our community, we have been obliged to diversity and equality, to liberty and freedom, and to truth and reason. Therefore, in the name of all glycoscientists who have contributed to this Thematic Series, I finally express the hope that the ethical standards that we have applied in our work will provide guidance and confidence, if our healthy world will once have to face the challenges of distrust, fanaticism and hate, as it is currently the case in many areas of this planet.

Thisbe K. Lindhorst

Kiel, October 2014
